# Portal venous gas detected by ultrasound examination in emergency department in a patient with acute abdominal pain

**DOI:** 10.1186/2036-7902-7-S1-A11

**Published:** 2015-03-09

**Authors:** A Oviedo-García, M Algaba-Montes, J Lopez-Libano, JM Alvarez-Franco, N Diaz-Rodriguez, A Rodriguez-Lorenzo

**Affiliations:** 1 Emergency Department, Valme Hospital, Seville, Spain; 2 Critical Care Department, Miramar Hospital, Mallorca, Spain; 3 Emergency Department, IB-Salut, Ibiza, Spain; 4 Primary Care, Barbadás Primary Care Center, Ourense, Spain; 5 Radiology Department, Perpetuo Socorro Hospital, Vigo, Spain

## Background

Intestinal pneumatosis (IP) defined as the presence of gas within the bowel wall and the presence of gas in the portomesenteric vein complex, a rare clinical condition that are typically associated with intestinal ischemia (II) and a fatal outcome.

## Objective

We present a case of IP, diagnosed at ED, through the use of US scanning used by EP.

## Patients and methods

A patient with abdominal pain, with a final diagnosis of a II assessing US, performed by EP.

## Results

82 year old woman, active and independent, who came to the ER with abdominal pain from 12 hours, which started about 2 hours after dinner. The patient presented malaise, affected by pain, hypotensive and tachycardic. Given the general malaise the patient underwent an abdominal ultrasound scan at the bedside of the patient showed many small echogenic mobile pictures that moved through the portal vein and its branches, and in the left hepatic lobe level we saw also multiple linear echogenic pictures in the portal branches with posterior acoustic shadows. Suspecting IP and gas in the abdominal venous complex portomesenteric urgent contrast CT was made, which confirmed the diagnosis.

## Conclusion

CT and US are the most commonly used imaging modalities in patients with acute abdomen and even if CT represents the gold standard in the evaluation of patients with II. However, there are some disadvantages associated with this technique, such as radiation exposure, potential nephrotoxicity and the risk of an allergic reaction to the contrast agents. Thus, not all patients with suspected bowel ischaemia can be subjected to these examinations. Despite its limitations, bedside ultrasound performed by EP could constitutes a good imaging method as first examination in acute settings of suspected mesenteric ischemia.

Ultrasonography by EP, can be a useful tool in cases with serious diseases. Incorporate ultrasound in the ER lowers overall service times, since the EP is more effective, efficient and dynamic management "time-dependent" emergency, providing greater clinical patient safety.

## Informed consent

The study was conducted in accordance with the ethical standards dictated by applicable law. Informed consent was obtained from each owner to enrolment in the study and to the inclusion in this article of information that could potentially lead to their identification.
